# Excellent spin transport in spin valves based on the conjugated polymer with high carrier mobility

**DOI:** 10.1038/srep09355

**Published:** 2015-03-23

**Authors:** Feng Li, Tian Li, Feng Chen, Fapei Zhang

**Affiliations:** 1 High Magnetic Field Laboratory, Chinese Academy of Sciences, Hefei 230031, China

## Abstract

Organic semiconductors (OSCs) are characteristic of long spin-relaxation lifetime due to weak spin-orbit interaction and hyperfine interaction. However, short spin diffusion length and weak magnetoresistance (MR) effect at room temperature (RT) was commonly found on spin valves (SVs) using an organic spacer, which should be correlated with low carrier mobility of the OSCs. Here, N-type semiconducting polymer P(NDI2OD-T2) with high carrier mobility is employed as the spacer in the SV devices. Exceedingly high MR ratio of 90.0% at 4.2 K and of 6.8% at RT are achieved, respectively, via improving the interface structure between the polymer interlayer and top cobalt electrode as well as optimal annealing of manganite bottom electrode. Furthermore, we observe spin dependent transport through the polymeric interlayer and a large spin diffusion length with a weak temperature dependence. The results indicate that this polymer material can be used as a good medium for spintronic devices.

One of basic spintronic devices is spin valve (SV), which consists of two ferromagnetic (FM) electrodes separated by a non-magnetic spacer. Giant magnetoresistance (GMR) effect at low temperature has been demonstrated on the vertical SV devices employing small molecules such as tris(8-hydroxyquinoline) aluminum (Alq_3_), fullerene (C_60_) and rubrene as the spacer^1^,^2^,^3^,^4^,^5^,^6^,^7^,^8^,^9^,^10^,^11^. In parallel, some π-conjugated polymeric materials such as poly (3-hexylthiophene) (P3HT) and DOO-PPV were also applied successfully in the SV structures^1^,^12^,^13^,^14^. Such polymer devices possess the advantage of low-cost fabrication and mechanical flexibility^15^.

However, although characteristic of long spin-diffusion time *t*_S_, organic semiconductors (OSCs) exhibit rather short spin-diffusion length *λ*_S_ (usually in the range of 10–50 nm) due to low carrier mobility of these materials^1^,^16^. According to the relationship between *λ*_S_ and *t*_S_, which can be expressed as 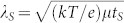 (*μ*: carrier mobility, *T*: temperature and *k*: Boltzmann constant)^15^, it intuitively suggests the choice of organic materials of favorable energy level and high carrier mobility to achieve longer spin diffusion length. Furthermore, although significant MR effect at RT was found on organic spin tunnel junctions^17^,^18^,^19^. Commonly the SV devices with thicker organic layer (>15 nm) show a remarkable decay of the MR below RT^2^,^3^,^4^,^5^,^10^,^11^, which was attributed to the decay of λ_S_ and/or spin polarization of the FM electrode with the temperature^1^,^2^,^3^,^8^,^20^. Only recently a large MR ratio of 4–6% have been achieved on the device structures of C_60_ (or bathocuproine) as a spacer between the FM Fe_3_O_4_ (or NiFe) and Co electrode^6^,^7^,^9^. Meanwhile, since the softness nature of the OSCs, the deposition of top FM electrode usually leads to the formation of an “ill-defined” layer in organic spacer, which deteriorates seriously the MR performance of the organic SV^1^,^2^,^3^,^13^,^21^. To get deeper insight on these issues, it will be of much significance to explore new organic materials for the SV application and to determine how material parameters such as film structure, carrier transport (and charge trap states) as well as the FM/organic interface are correlated with spin transport of the SV devices.

In this work, we report at the first time on the SV devices based on an air stable N-type semiconducting polymer poly{[N,N′-bis(2-octyldodecyl)-1,4,5,8-naphthalenedicarboximide)-2,6-diyl]-alt-5,5′-(2,2′-bithio-phene)} (P(NDI2OD-T2)^22^ by a simple solution process. We study carrier/spin injection and transport in the devices. By modifying the FM electrode/polymer interface to improve the spin injection/detection efficiency, a MR ratio of *ca.* 90% at 4.2 K and 6.8% at RT (one of the highest MR effect reported to date) is achieved, respectively. On the other hand, P(NDI2OD-T2) is found to exhibit a large diffusion length at both low temperature and RT, which indicate that this material can be used as a ideal medium for spin-dependent transport. The work will strongly encourage us to look for more OSC materials with good carrier/spin transport properties for spintronics applications, as well as to develop high performance organic SV devices.

## Results

### Materials and device fabrication

**Figure 1:**
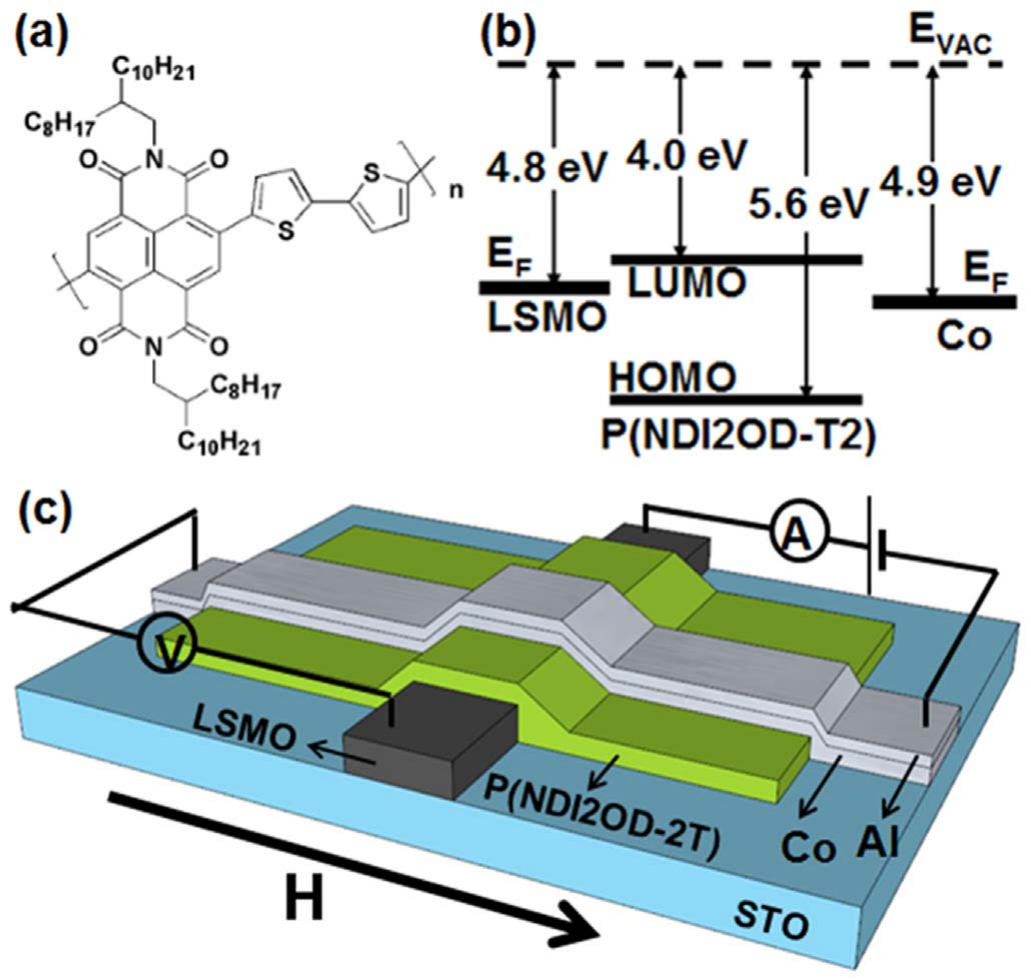
Structure of polymeric spin valve devices. (a) Chemical structure of the polymer P(NDI2OD-T2). (b) Schematic rigid energy diagram of the organic device which shows the Fermi levels (E_F_) of two ferromagnetic electrodes (LSMO and Co), the highest occupied molecular orbital (HOMO) and the lowest unoccupied molecular orbital (LUMO) levels of P(NDI2OD-T2). (c) Schematic diagram of the device structure of “LSMO/P(NDI2OD-T2)/Co/Al”.

Figure 1a shows chemical structure of P(NDI2OD-T2). The polymer exhibits high electron mobility of 0.20–0.85 cm^2^/Vs in organic thin-film transistors^23^. Surprisingly this material shows a weak long-range order however a low energy disorder within the film^24^,^25^. Furthermore as found from synchrotron X-ray scattering, the polymer takes a largely face-on molecular packing (π-stacking direction normal to the substrate)^25^. These characters lead to enhanced carrier mobility (up to 6.0 × 10^−3^ cm^2^/Vs) in vertical diode structures^26^. Therefore it is naturally expected that these structural characteristics should be favorable to spin transport of carriers, for example to enhance spin diffusion length in the vertical spin valves^27^. Besides, P(NDI2OD-T2) has a low-lying lowest unoccupied molecular orbital (LUMO) level of 4.0 eV, matched with the Fermi levels (E_F_) of common FM metals (as shown in Fig. 1b), which makes a ready carriers injection from the electrodes. Finally, the polymer is also very stable in air and the devices can operate in ambient condition^22^,^23^. Therefore we expect that it will act as a suitable spacer in organic SVs.

The polymeric SV device with the structure of SrTiO_3_ (STO) substrate/La_2/3_Sr_1/3_MnO_3_ (LSMO)/P(NDI2OD-T2)/Co/Al is shown schematically in Fig. 1c, the detailed fabrication processes are found in Method Section. The LSMO electrodes deposited exhibit a relatively high Curie temperature (*T*_c_) of 365 K (see Supplementary Fig. S1 online). The polymeric interlayer with varied film thickness (30–120 nm) was spin-coated on the LSMO electrode. P(NDI2OD-T2) is highly soluble in common organic solvents and thus the homogenous and smooth film was formed from spin-coating of dichlorobenzene solution. The 10 nm Co film was thermally evaporated as top FM electrode. A low-power evaporation process was applied to prevent film damage of P(NDI2OD-T2) from heat irradiation during Co deposition. In addition, a 1.5 nm AlO_x_ interlayer was inserted between top Co electrode and the polymeric spacer for some of the samples (see Method section). Such a thin layer was found to enhance spin polarization injection into organic spacer of the SVs in previous investigations^3^,^6^,^8^.

### Film microstructure and Co/P(NDI2OD-T2) interface

Specular X-ray diffraction pattern (Supplementary Fig. S2 online) shows no distinct diffraction peaks on the P(NDI2OD-T2) film, indicating a seemingly amorphous-like structure. However, high-intensity synchrotron-based grazing incidence X-ray diffraction (GIXRD) patterns (see Fig. S2) display a wide and weak π-stacking reflection along the out-of-plane direction as well as multiple orders of lamellar stacking and chain backbone repeat peaks along the in-plane direction. It confirms a predominantly face-on packing and high degree of in-plane ordering within the film, in consistent with the previous reports^25^,^26^. From atomic force microscopy (AFM) measurements (typical surface topographic image is shown in Supplementary Fig. S3), the film of P(NDI2OD-T2) displays a homogeneous surface with fiber-like morphology. The surface root mean squared (RMS) roughness is found to be only 0.45 ± 0.02 nm and independent of film thicknesses. Low surface roughness and high uniformity of the film will be conducive to fabricate the organic SV devices with high quality and high operation stability.

**Figure 2:**
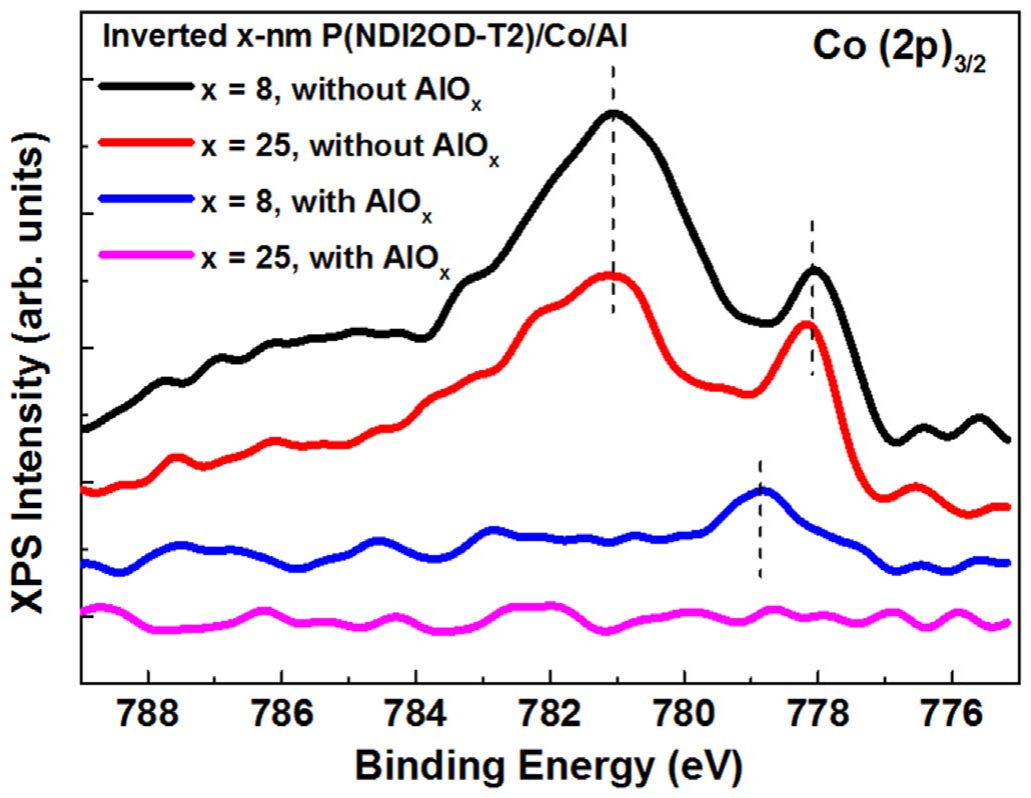
Structural properties of the Co/polymer interfaces. X-ray photoelectron spectra (XPS) of Co (2p)_3/2_ at the inverted P(NDI2OD-T2)/Co and inverted P(NDI2OD-T2)/AlO_x_/Co interfaces. The take-off angle (angle between the direction of the detected electrons and the surface of the sample) is 85°. The dashed lines in the diagram are visual guides.

The formation of sharp FM/organic interface is vital for high-performance organic SVs. In order to investigate interface reaction and inter-diffusion during Co deposition, the “inverted” interfacial structures were prepared and investigated by means of the X-ray photoelectron spectroscopy (XPS) measurements^8^,^28^. Figure 2 shows the Co (2p)_3/2_ core-level spectra of the P(NDI2OD-T2) layers on the Co film (the inverted Co/P(NDI2OD-T2) interface) as well as on the 1.5-nm AlO_x_/Co bilayer (the inverted Co/AlO_x_/P(NDI2OD-T2) interface) (see Method section), respectively. For the former circumstance, a weak but appreciable Co 2p peak is observed on the samples with both 8-nm and 25-nm P(NDI2OD-T2) layers. The wide peak centered at the binding energy (BE) of 781.2 eV and the peak with lower BE of 778.1 eV, comes from the contribution of the oxide CoO_x_ component (also some amount of Co-N component, see the deconvoluted spectra in Supplementary Fig. S4 online) and of metallic cobalt^29^, respectively. The component of CoO_x_ dominates over that of the metallic Co, indicating a strong interface reaction, which should originate from the interaction between Co atoms and carbonyl (C = O) group on the P(NDI2OD-T2) molecules during Co deposition. From the analysis on intensity decay of Co (2p)_3/2_ with the P(NDI2OD-T2) thickness (see Supplementary Note), it indicates that Co penetration into the P(NDI2OD-T2) layer occurs during Co deposition and most of cobalt atoms penetrated should be concentrated on the region near the Co/polymer interface. It should be mentioned that metal inter-diffusion is much less serious here compared to the other FM/organic interfaces, in which an “ill-defined” layer of up to 25 nm was observed on the Co/Alq_3_ system due to cobalt inclusion in the Alq_3_ layer^21^.

For the inverted Co/AlO_x_/P(NDI2OD-T2) interface, the Co (2p)_3/2_ peak for the sample covered with a 8 nm polymeric layer becomes drastically weaker (intensity decay by a factor of 8) with respect to the interface without the AlO_x_ barrier. Furthermore, the CoO_x_ component disappears meanwhile a peak at the BE of 778.7 eV becomes dominant which position is close to the component of metallic Co. The Co (2p)_3/2_ peak becomes no longer discernable for the sample with a 25-nm P(NDI2OD-T2) layer. These results clearly indicate that an AlO_x_ barrier can effectively prevent interfacial reaction as well as cobalt penetration so that a “sharp” Co/polymer interface forms during Co deposition. Therefore, possible presence of pinholes and filament conduction channels within the P(NDI2OD-T2) layer could be excluded based on remarkably reduced cobalt inclusion and on the homogeneous film morphology.

### Spin transport of P(NDI2OD-T2) devices

**Figure 3:**
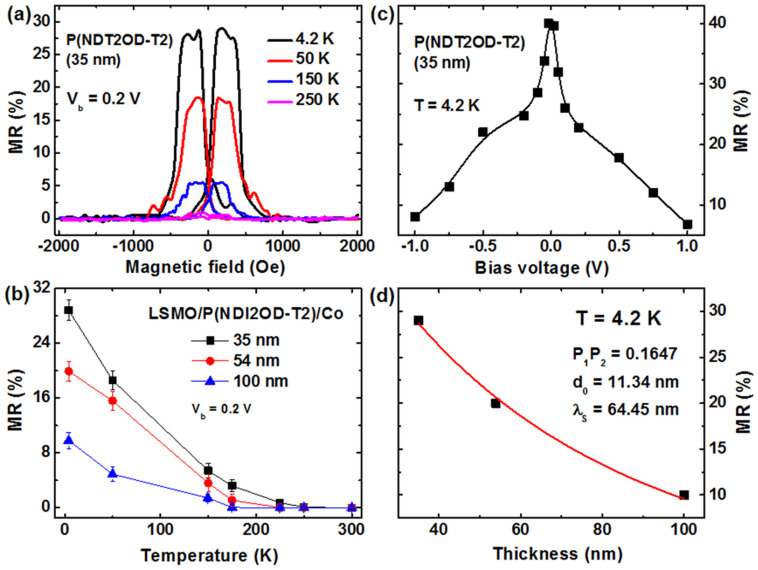
Spin transport properties of the polymeric spin valve devices. (a) MR curves of the SV device with a 35 nm P(NDI2OD-T2) interlayer measured at different temperatures. The bias voltage was fixed at 0.2 V for the MR measurement (b) The temperature dependent MR data of the polymeric SVs with different P(NDI2OD-T2) thicknesses. Error bar represents the standard deviation of MRs and are made based on the scatter in the MR data from three devices at each thickness. (c) Bias-voltage dependence of the MR values for the polymer SV with the 35 nm P(NDI2OD-T2) interlayer measured at 4.2 K. (d) MR ratios of the SVs as a function of the P(NDI2OD-T2) thickness, as well as the fitting for MR data by the modified Julliere formula (Equation (1)) at 4.2 K.

Figure 3a shows typical MR traces for the polymeric SV with a 35 nm P(NDI2OD-T2) interlayer. A high positive MR value of nearly 30% is achieved at 4.2 K but the magnitude of the MR signal decays steeply with the increased temperature. No MR effect is observed above 250 K. It should be mentioned that all of the fabricated P(NDI2OD-T2)-based devices exhibit a junction resistance larger than 10 kΩ, which is about two orders of magnitude higher than the resistances of the LSMO and Co electrodes, thus the MR signal should not be attributed to anisotropic magnetoresistance (AMR) effect of the electrodes. For the devices of the thicker P(NDI2OD-T2) interlayers (see Supplementary Fig. S5 online), the MR signals decays remarkably with the increased polymer thickness. However, a reasonably high MR of 9.8% is still achieved at 4.2 K even on the SV device of a 100 nm P(NDI2OD-T2). Figure 3b summarizes the MR data as a function of the measurement temperature for the SV devices with different interlayer thicknesses. All of the devices exhibit a steep decrease of MR ratio with increased temperature and no MR effect was observed at RT. Such temperature dependence of the MR is consistent with the results previously reported on the organic SVs^2^,^3^,^8^,^10^, which was commonly attributed to the weakened surface spin polarization of the LSMO electrode with increased temperature or spin polarization relaxation in the organic interlayer. This issue will be elaborated in detail below.

Bias-voltage dependence of the MR ratios for the SV devices with a 35-nm P(NDI2OD-T2) interlayer was studied as shown in Fig. 3c. In spite of fast decay with the bias-voltage, a reasonably large MR signal of *ca*. 8.0% is still observed at the bias-voltage of 1.0 V, which indicates good quality of our polymeric SVs. It is worthy to note that a high MR of 40% at 4.2 K is achieved when the polymeric devices were measured at a small bias of 0.02 V. Moreover, a clear asymmetry of the MR curve with the bias polarity is also found, which may origin from the difference on work function of the employed LSMO and Co electrodes^2^,^13^,^30^.

### Charge carrier transport of P(NDI2OD-T2) devices

**Figure 4:**
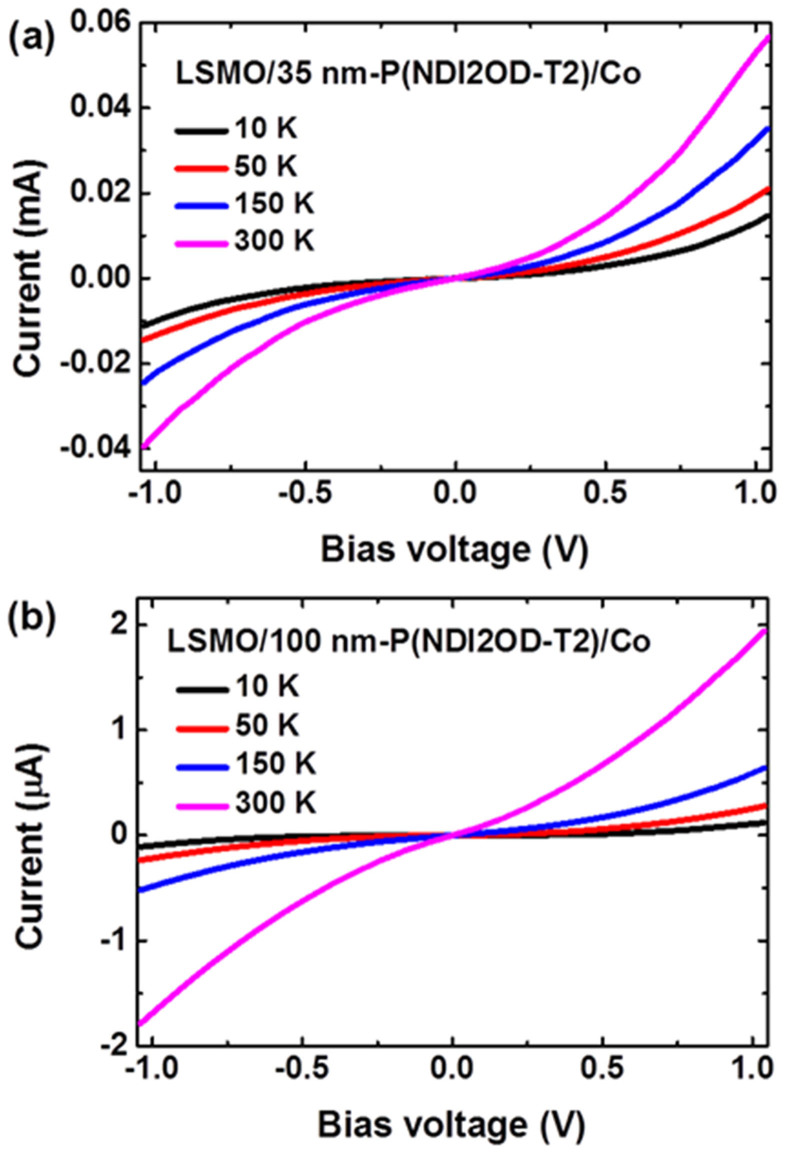
Carrier transport properties of polymeric spin valves. The current–voltage (*I–V*) curves at different temperatures for the devices with a P(NDI2OD-T2) interlayer of (a) 35 nm and (b) 100 nm, respectively.

In order to understand the mechanism of spin-dependent transport in the P(NDI2OD-T2)-based SV devices, current-voltage (*I–V*) characteristics of the devices with the different thicknesses of the polymeric interlayer were measured at different temperatures. *I–V* curves of Fig. 4 show a clear non-linear nature. Furthermore, temperature dependence of *I–V* behaviour becomes remarkably stronger when the thickness of P(NDI2OD-T2) increases from 35 nm to 100 nm. In light of the criteria for distinguishing spin-dependent tunneling and injection^4^,^10^, such electrical behaviour, together with the MR characteristics, indicates that spin-polarized electrons inject from the electrodes and take hopping transport in the N-type P(NDI2OD-T2) interlayer. Electrons hop more facilely among the LUMO levels of adjacent molecules with π-stacking of the conjugated backbones along the vertical direction of the film.

However, at the moment it is still difficult to determine unambiguously which step, *i.e.* carrier injection at the polymeric/FM interface or carrier transport in bulk polymeric spacer, dominates spin-dependent transport behaviour of the P(NDI2OD-T2) devices. Steyrleuthner *et al* previously observed the injected limited current (*i.e.* the absence of the space charge limited current) in the electron-only diode structures of P(NDI2OD-T2) even employing low work function electrodes (for example Sm and Ca)^31^. Considering the large nominal injection barrier (*ca.* 0.8–0.9 eV) between the FM electrodes (LSMO and Co) and P(NDI2OD-T2), it is suggested that electron injection at the FM/polymer interface should be the main limiting factor for spin transport of our SV devices. Surely comprehensive investigations on interfacial dipole effect, interfacial states and density of trap states in the polymeric film should be performed to understand their impacts on carrier injection/transport processes of the P(NDI2OD-T2) devices^5^,^31^,^32^,^33^.

### Spin transport of P(NDI2OD-T2) devices with a AlO_x_ barrier

**Figure 5:**
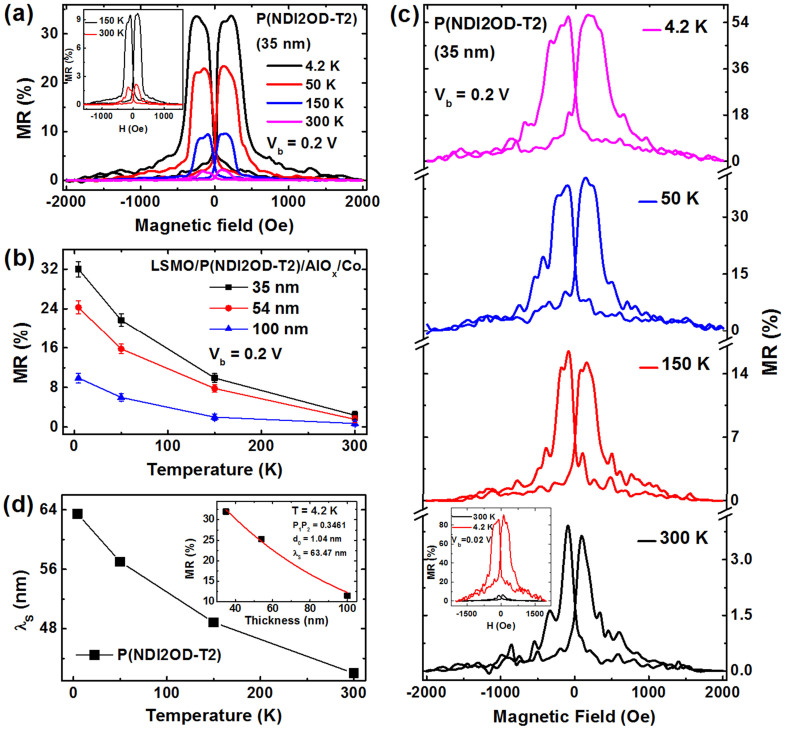
Spin transport properties of polymeric spin valves with an AlO_x_ barrier. (a) MR curves for the LSMO/P(NDI2OD-T2)/AlO_x_/Co device with a 35-nm P(NDI2OD-T2) interlayer at different temperatures. The inset shows the enlarged graphics for the MR curves at higher temperatures. (b) The temperature dependent MR data of the devices with different thicknesses of P(NDI2OD-T2). Error bar denote the standard deviation of MR for each thickness. (c) MR curves for the SV device of a 35-nm P(NDI2OD-T2) interlayer in which the bottom LSMO electrode was annealed at 1050°C before device fabrication. The inset shows the MR ratios of the same device measured at a low bias of 0.02 V. (d) The temperature dependence of spin diffusion length *λ*_S_ of the polymer P(NDI2OD-T2), obtained from the fitting of the MR data in Fig. 5b for the devices with different interlayer thicknesses. The inset shows the fitting of the MR values measured at 4.2 K by Equation (1).

A 1.5-nm AlO_x_ interfacial layer was introduced between the P(NDI2OD-T2) and the Co electrode to manipulate spin-polarized injection behaviour. Figure 5a shows typical MR curves of the SV device of a 35-nm polymer interlayer. The devices exhibit a slight enhancement of the MR effect at low temperatures (up to 33% at 4.2 K) with respective to those without an AlO_x_ barrier. Interestingly the magnitude of MR signal at higher temperature is remarkably enhanced and an appreciable MR ratio of 2.0% is achieved at RT (Inset of Fig. 5a). A similar improvement of MR ratio is also found on the SVs with the larger P(NDI2OD-T2) thickness, as shown in the summarized data in Fig. 5b and Supplementary Fig. S6. The results suggest that the improved performance should originate from the improved interface quality and thus enhanced spin injection efficiency at the polymeric/Co interface.

On the side of the LSMO electrode, recently Chen *et al* have reported that annealing at high temperature above 1000°C enhances surface spin polarization of the LSMO electrode and thus results in enhanced GMR effect at RT for the Alq_3_-based SVs^34^. In this work, the bottom LSMO electrode was annealed in oxygen at 1050°C for 6 h prior to the deposition of P(NDI2OD-T2). Figure 5c shows the MR traces of a SV device of 35-nm P(NDI2OD-T2) interlayer (measured at the bias of 0.2 V). MR value as high as 4.5% at RT and 58.5% at 4.2 K has been achieved for the device via optimal annealing of the LSMO electrode (also introducing an AlO_x_ barrier), respectively, an remarkable enhancement compared to the devices with the LSMO annealed at 800°C. Furthermore, when the device is applied with the bias of 0.02 V (the inset of Fig. 5c), the MR ratio reaches *ca.* 90.0% at 4.2 K and 6.8% at RT, respectively, one of the largest MR at RT reported to date^6^,^7^,^9^,^17^,^19^. These results indicate that the polymeric material P(NDI2OD-T2) possesses good spin transport properties and exhibits excellent MR behaviour on the SV devices when the good carrier/spin injection is fulfilled. Since the key role of the FM/polymer interface on device performance, the modification of interfacial structure and energy level alignment is necessary to further improve carrier and spin injection efficiency.

## Discussion

In order to elucidate the origin for high MR characteristics of the P(NDI2OD-T2) devices, we apply the modified Julliere formula^2^,^35^ to fit the MR results. Considering the decay of spin polarization of the injected carriers in the OSC spacer, the MR ratio will be given by
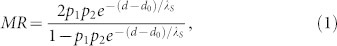
where *p*_1_ and *p*_2_ denotes the spin polarization of LSMO and Co, respectively, *d* is the polymeric interlayer thickness, *d*_0_ is the “ill-defined” layer in the polymeric spacer near the Co/polymer interface due to metal penetration^2^. Equation (1) was used to fit the MR data in Fig. 3(b) with three adjustable parameters of *p*_1_*p*_2_, *d*_0_ and *λ*_S_. Good agreement was obtained for the MR data at 4.2 K as shown in Fig. 3(d), with the following parameters: *p*_1_*p*_2_ = 0.1647, *d*_0_ = 11.34 nm and *λ*_S_ = 64.45 nm. The product of spin polarization of both FM electrodes *p*_1_*p*_2_ is much lower than the expected value considering high spin polarization of LSMO at low temperature, which should be mainly attributed to poor spin injection efficiency from the Co electrode into P(NDI2OD-T2). On the other hand, a large spin diffusion length *λ*_S_ (*ca.* 64.5 nm) is obtained and it shows just a slight decrease (*ca.* 49 nm) at 150 K (see Supplementary Fig. S7 online). Importantly, the “ill-defined” layer *d_0_* of 11–13 nm is extracted by fitting the polymer thickness dependence of MR at different temperature, which reflects a strong interfacial reaction and cobalt penetration during the deposition of metal electrode, in consistent with above XPS results on the Co/P(NDI2OD-T2) interface.

The fitting of the MR data was also made for the SV devices with an AlO_x_ barrier. The inset of Fig. 5(d) shows the fitting result at 4.2 K with the following parameters: *p*_1_*p*_2_ = 0.3461, *d*_0_ = 1.04 nm and *λ*_S_ = 63.47 nm. Compared to the devices without the AlO_x_ layer, the enhanced product of spin polarization of LSMO and Co (*p*_1_*p*_2_) indicates improved spin injection efficiency. Furthermore, a steeply reduced thickness of “ill-defined” layer *d*_0_ is found. The similar *d*_0_ value in the range of 1.0–1.6 nm is also estimated from the fitting for the MR values at higher temperature (see Supplementary Fig. S8). This result is in good agreement to the XPS measurements shown in Fig. 2. It clearly demonstrates that the introduction of a thin AlO_x_ barrier prevents effectively Co penetration at polymer/Co interface during metal deposition and thus enhances remarkably spin dependent injection at the FM/polymer interface.

On the other hand, it is found from the fitting that spin diffusion length *λ*_S_ at 4.2 K keeps nearly unchanged for the P(NDI2OD-T2) devices no matter whether the AlO_x_ barrier is introduced. Furthermore, the *λ*_S_ exhibits only a slow decay with the temperature and keeps reasonably high value of 41.87 nm at RT as shown in Fig. 5d, which is unusual among the OSCs commonly used in spintronics. Usually most of these materials exhibit a short and strongly temperature dependent *λ*_S_^2^,^3^,^11^,^12^,^13^. It indicates that the polymeric semiconductor P(NDI2OD-T2) can be utilized as an excellent medium for spin dependent transport.

The outstanding spin dependent transport should be correlated with the high short-range order as well as face-on molecular packing of the P(NDI2OD-T2) film^25^,^26^. The delocalization of electrons at nanoscale length along the out-of-plane direction, originating from strong inter-molecular π-stacking, enable the spin- polarized carriers make the longer-range hops to maintain their initial spin orientation. Good spin transport will be also associated to extremely low degree of energetic disorder (e.g. defects and trap states)^24^,^36^, which can lead to a decreased spin-flip sites^37^. These two factors will contribute to weaken the spin scattering effect especially at the higher temperature regime where the density of carriers and hopping rates increase drastically, therefore to achieve a high *λ*_s _at RT. This is in agreement with the prediction by Bobbert *et al* in which the *λ*_s_ and mobility is enhanced with the decreased (energetic) disorder strength in the disordered OSCs^38^. Experimentally, Mooser et al observed a clear scaling of the MR value of the OSVs with the bulk mobility of TIPS-pentacene spacer^11^, that is, the MR and mobility decrease concurrently with decreasing temperature, which was attributed to the reduced *λ*_s_ due to low carrier mobility at low temperature. Recently extraordinarily large *λ*_S_ of 200 nm has been observed by ferromagnetic resonance spin pumping technique in polymeric PBTTT^39^ (It will be to note that due to the minimized carrier injection effect for this technique to measure the *λ*_S_, a larger value of *λ*_S_ could be expected compared to that obtained from spin valves.) while a large *λ*_S_ in regio-regular (RR)-P3HT was found at low temperature from the MR measurement of spin valves^40^. Both of conjugated polymers exhibit ordered semi-crystalline structure with two-dimensional lamellar structure as well as high field-effect (hole) mobility^41^,^42^. Furthermore the *λ*_S_ was found to be remarkably larger for RR-P3HT compared to regio-random (RRa)-P3HT^43^. All of these evidences indicate the importance role of film structural order on spin dependent transport.

It will be pointed out that, weak temperature dependence of spin diffusion length λ_S_ provides valuable implication on main source of spin relaxation within P(NDI2OD-T2). Carrier mobility μ of P(NDI2OD-T2), reported on both the diode structures and the FET devices, exhibits a thermal activation behavior. Therefore spin relaxation time t_S,_ extracted based on the relation 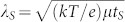, should decreases drastically with increasing temperature. It indicates that the spin-orbit coupling (SOC) should be main source for spin relaxation process in this polymer, similar to the cases on the other semiconducting polymers like PBTTT.

Our studies show the strong interplay between film microstructure and magneto-transport, which is also evidenced from the literature^7^,^11^,^44^. Finer controlling of molecular orientation and packing as well as of the degree of order (e. g. molecular aggregates and crystallinity) within the film of P(NDI2OD-T2) will be of much interest to study their influence on spin dependent transport and flip process as well as to further improve the λ_s_ and MR performance. Furthermore, our work will also encourage one to explore the class of conjugated polymer systems with the similar “donor-accepter” type molecular structure and similar film structural characteristics as P(NDI2OD-T2)^27^, all of which display high carrier mobility, for the spintronic application.

In conclusion, we have prepared the SVs based on semiconducting polymer P(NDI2OD-T2). The studies of device characteristics reveal that the MR effect originates from spin-polarized carrier injection into and consequently transport through P(NDI2OD-T2). The improvement of interface properties via introducing a thin AlO_x_ barrier at the Co/polymer interface, together with optimal annealing of bottom LSMO electrode, leads to an exceedingly high MR ratio at both low temperature and RT. Furthermore, it is found that P(NDI2OD-T2) has the large spin relaxation length with a weak temperature dependence. Outstanding spin transport properties and SV performance make P(NDI2OD-T2) a promising material for the spintronic application. This work will encourage us to explore more novel organic materials for the development of high-performance spintronic devices, and will be of much significance for both scientific research and future industrial application in organic electronics.

## Methods

### Sample fabrication

The 100 nm LSMO electrodes were deposited on the SrTiO_3_ (100) substrates using pulsed laser deposition (PLD) method through a shadow mask to form the 1.0 mm wide stripes, followed by annealing at 800°C or 1050°C for 6 h in flowing pure oxygen. P(NDI2OD-T2) was purchased from Polyera Corporation (Activink™ N2200). After cleaning the LSMO substrate using acetone, a polymeric P(NDI2OD-T2) layer was deposited by spin-coating from the 1.0% (*w*/*w*) dichlorobenzene solution and then annealed at 110°C for 5 h in the glove-box to remove the remnant solvent within the film. The thickness of the polymeric layer was controlled in the range of 25–150 nm via varying the spin-coating rate (from 1000 rpm to 5000 rpm) and measured by the surface profiler and the field-emission scanning electron microscopy. Finally, a 10-nm Co film was thermally evaporated as top ferromagnetic electrode and subsequently capped with a 30 nm Al protection layer using a shadow mask. The cross-bar junction area was about 0.5 × 1.0 mm^2^. For some of the SV devices, a *ca.* 1.0-nm Al layer was deposited on the top of the P(NDI2OD-T2) layer by thermal evaporation and subsequent oxidized by exposure in air to form a 1.5-nm AlO_x_ layer prior to the Co electrode.

### Experimental setup

The crystallinity of the P(NDI2OD-T2) film was identified by specular scan X-ray diffraction (XRD) using the Rigaku-TTR3 X-ray diffractometer with Cu *Kα* (*λ* = 1.5406 Å) radiation as well as by the synchrotron based 2-dimensional (2D) grazing incidence X-ray diffraction (GIXRD). The GIXRD measurement was performed at Shanghai Synchrotron Radiation Facility (SSRF) on the beam line BL14B with the photon energy of 8.0 keV. The morphology of the P(NDI2OD-T2) layer was characterized with a Veeco MultiMode (Nanoscope V) atomic force microscopy (AFM) in tapping mode. The interface quality of the Co/P(NDI2OD-T2) were investigated by X-ray photoelectron spectroscopy (XPS) using monochromatized Al (*K_α_*) X-ray at *hν* = 1486.6 eV. For the investigation of the Co/P(NDI2OD-T2) interface, firstly, an 8 nm (or 25 nm) P(NDI2OD-T2) film was spin coated on a cleaned Si substrate (a dilute (0.2%) solution was employed for the preparation of the 8-nm film) followed by depositing a 10 nm Co film or a 1.5 nm AlO_x_ followed by 10 nm Co. Then the sample was taken from vacuum and an ex situ peel-off technique was applied to turn over the Co/P(NDI2OD-T2) or Co/AlO_x_/P(NDI2OD-T2) sample. Therefore an inverted interface (that is the P(NDI2OD-T2)-on-Co) sample was prepared with an intact interface morphology.

Magnetic properties of the ferromagnetic electrodes were measured by a Quantum Design Superconducting Quantum Interference Device (SQUID) magnetometer. The magnetoresistance (MR) of the polymeric SVs was measured in a Quantum Design Physical Property Measurement System (PPMS) under an external in-plane magnetic field from RT to 4.2 K using the standard four-probe method. The MR response was calculated using the expression MR = (*R*_AP_ − *R*_P_)/*R*_P_ × 100%, where *R*_AP_ and *R*_P_ is the resistance of the anti-parallel and parallel magnetization configuration of two electrodes. The devices current-voltage (*I–V*) measurements were performed using a Keithley 2612A source-measure unit.

## Author Contributions

F.Z. and F.L. conceived and designed the experiments, analyzed the data. F.L. performed the fabrication and characterization of the SV devices, conducted the AFM measurement. T.L. carried out the XPS and XRD experiments. F.C. performed the growth and magnetic measurement of the LSMO film. F.L. and F.Z. wrote the manuscript. F.Z. supervised the entire work. All authors participated in discussion on the manuscript.

## Supplementary Material

Supplementary Information Supplementary Information
