# Supplementary material for: Excellent spin transport in spin valves based on the conjugated polymer with high carrier mobility
Source: Sci Rep. 2015 Mar 23;5:9355. doi: 10.1038/srep09355 (PMC4369752; doi:10.1038/srep09355)
Supplement: Supplementary Information [file srep09355-s1.pdf]

Supplementary information

Excellent spin transport in spin valves based on the conjugated polymer with high carrier mobility

Feng Li, Tian Li, Feng Chen, and Fapei Zhang*

High Magnetic Field Laboratory, Chinese Academy of Sciences, Hefei 230031, China

Email: fzhang@hmfl.ac.cn

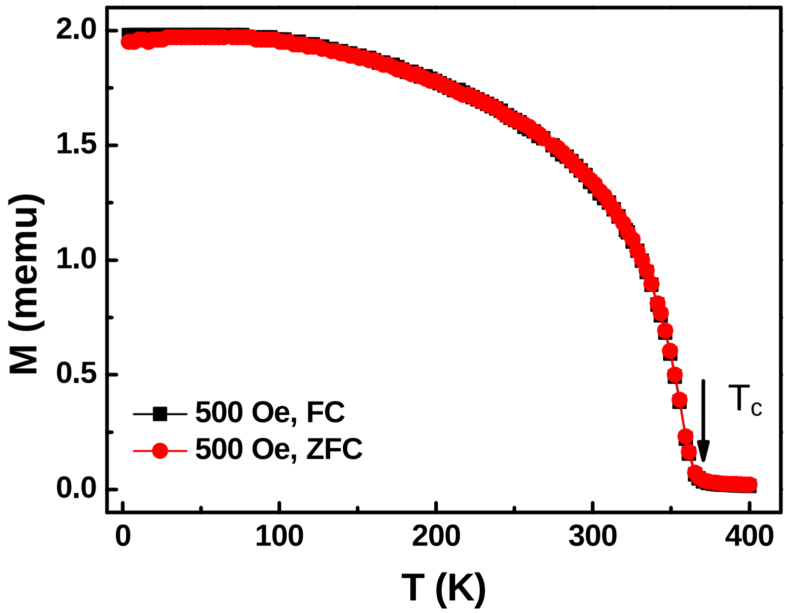

Figure S1 | Magnetization-temperature (M-T) curves of a 100-nm LSMO film growth on the STO (100) substrate. The sample was applied by the magnetic field of 500 Oe parallel to the film surface. The Curies temperature (*T*c) was determined as *ca*. 365 °C. FC: field-cooled, ZFC: zero-field-cooled.

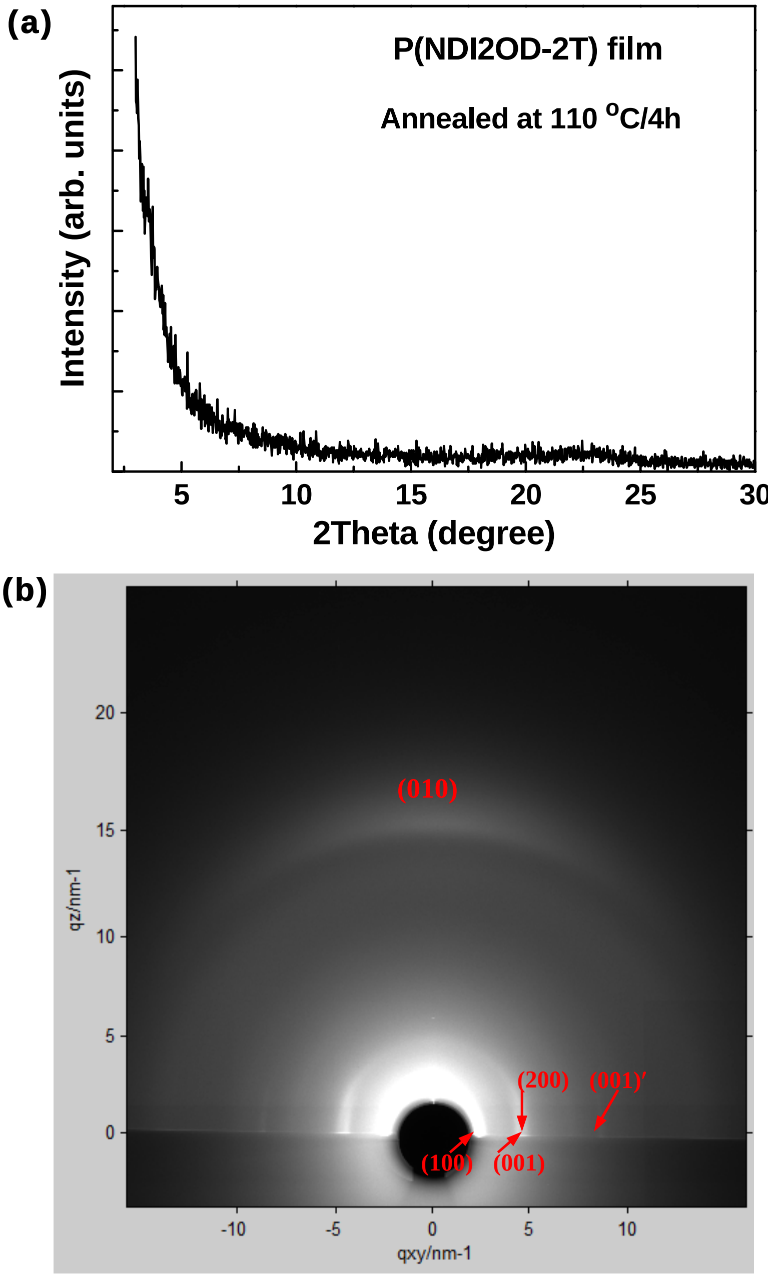

Figure S2 | Structural characterization of the P(NDI2OD-T2) films. (a) Specular scan X-ray diffraction (XRD) pattern and **(b)** synchrotron-based 2D grazing incidence X-ray diffraction (GIXRD) pattern of a 100-nm P(NDI2OD-T2) film deposited on the SiO_2/Si substrate. The sample was annealed at 110 °C for 4 h in the nitrogen atmosphere. The incidence angle of X-ray in GIXRD experiment is 0.15°. The GIXRD patterns display a wide π-stacking reflection (010) along the out-of-plane (q_z) direction as well as multiple orders of lamellar stacking (h00) peaks and chain backbone repeat (00k) peaks along the film in-plane (q_{xy}) direction.

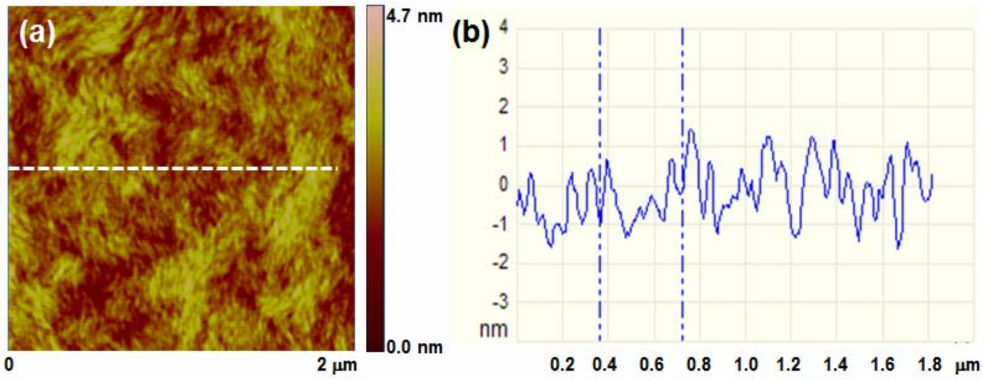

Figure 3S | Film morphology of a polymeric interlayer. (**a**) Tapping-mode AFM image of a 54 nm P(NDI2OD-T2) film spin-coated on the LSMO/STO substrate. (**b**) The profile along the line highlighted in (**a**).

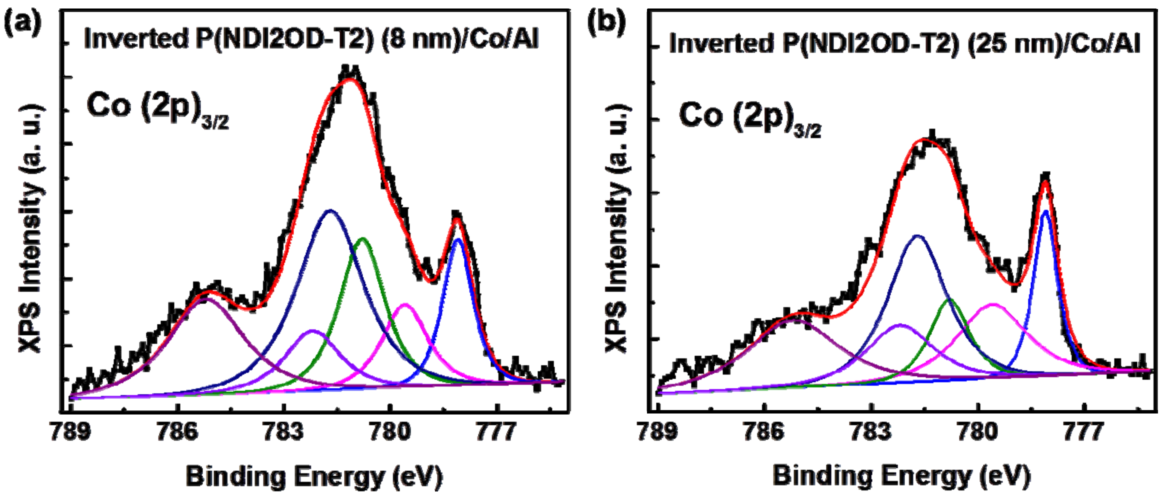

Figure S4 | The deconvolution of the Co (2p)3/2 XPS spectra. The spectra were obtained from the inverted P(NDI2OD-T2)/Co interfaces with the polymer thickness of 8 nm (Figure a) and 25 nm (Figure b), respectively. The solid black circles indicate the measured XPS data whereas the red curves are the fitted lines obtained by superposition of individual components (blue curve: metallic Co, magenta curve and purple curve: Co_3O_4, olive curve: N-Co bonding, navy curve: CoO, and violet curve: $Co(OH)_2$). High intensity of the CoO_x components with different valence states as well as the Co-N component indicates a strong interfacial reaction between cobalt and P(NDI2OD-T2).

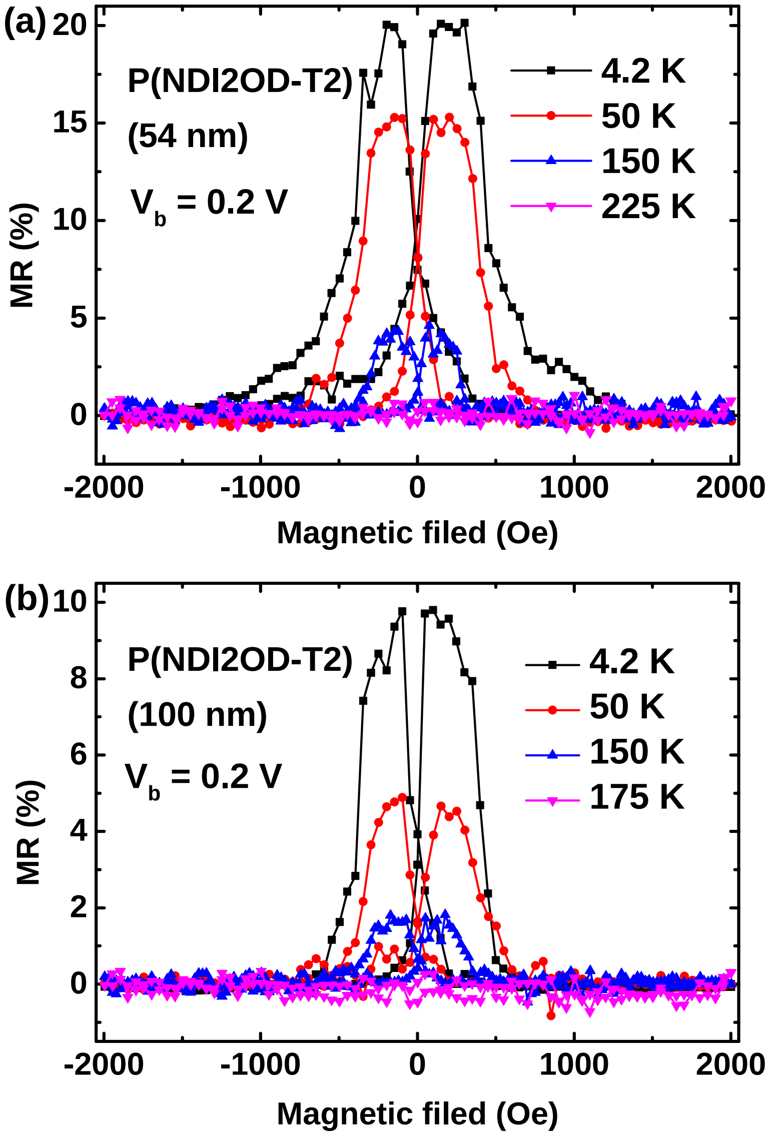

Figure S5 | MR curves of polymeric spin valve devices. The LSMO/P(NDI2OD-T2)/Co/Al devices with the P(NDI2OD-T2) interlayer of 54 nm **(a)** and 100 nm (**b**) measured at different temperatures

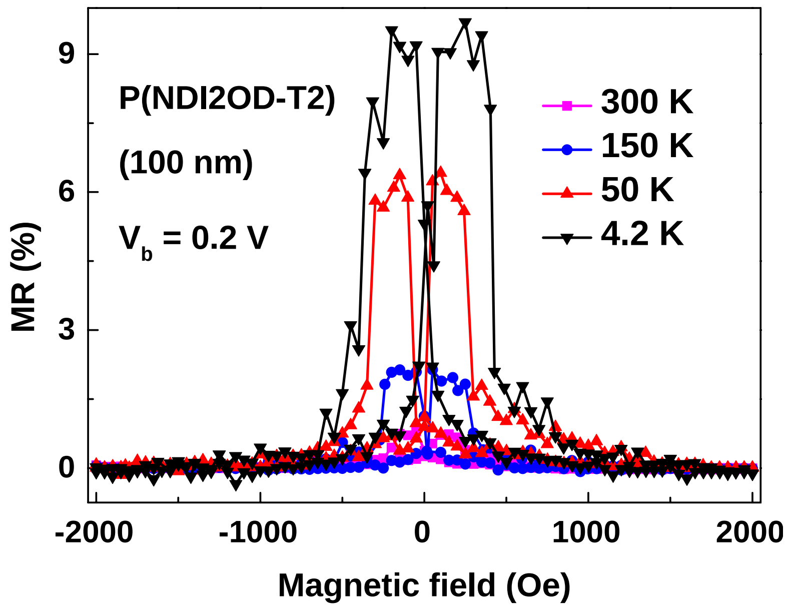

Figure S6 | Typical MR curves for the spin valve device of a 100-nm P(NDI2OD-T2) with a AlO_x barrier.

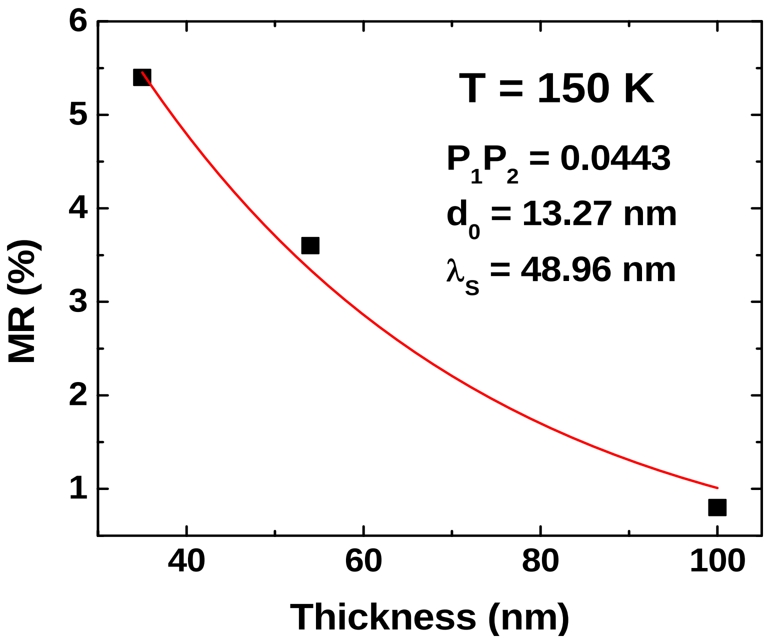

Figure S7 | The fitting of the P(NDI2OD-T2) thickness dependent MR data for the spin valve devices measured at 150 K by the modified Julliere formula.

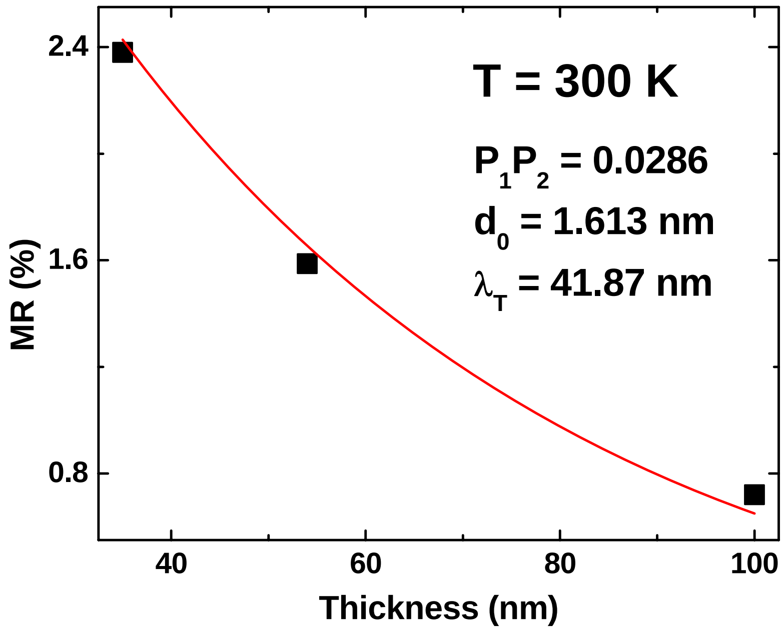

Figure S8 | The MR data as a function of the P(NDI2OD-T2) thickness measured at RT for the spin valves with a AlO_x barrier, as well as the fitting by the modified Julliere formula.

Supplementary Note | The analysis on the intensity decay of Co (2p$_{3/2}$) with the P(NDI2OD-T2) thickness. The elastic mean-free path of electrons emitted from the Co 2p level is *ca.* 2.1 nm (Ref: Tanuma, S., Powell, C. Calculations of Electron Inelastic Mean Free Paths. *Surf. Interface Anal.* **21**, 165 (1994)). Therefore the Co 2p peak from the Co layer fully covered by a 25-nm P(NDI2OD-T2) should decays to 0.042% of the intensity (and thus experimentally indiscernible) of an uncovered Co film. However, the measured Co 2p intensity still keeps 4.6% of the intensity for the bare Co. For the 8 nm P(NDI2OD-T2)/Co system the Co 2p intensity is enhanced by a factor of 3. Therefore it indicates cobalt penetration into the P(NDI2OD-T2) layer during Co deposition. Because of fast decay of Co 2p intensity with the thickness of P(NDI2OD-T2) layer covered, most of Co atoms penetrated should be concentrated on the region near the polymer/electrode interface.
